# Telomerase Reverse Transcriptase Increases Proliferation and Lifespan of Human NK Cells without Immortalization

**DOI:** 10.3390/biomedicines9060662

**Published:** 2021-06-09

**Authors:** Maria A. Streltsova, Maria O. Ustiuzhanina, Eugene V. Barsov, Sofya A. Kust, Rodion A. Velichinskii, Elena I. Kovalenko

**Affiliations:** 1 Shemyakin & Ovchinnikov Institute of Bioorganic Chemistry, Russian Academy of Sciences, ul. Miklukho-Maklaya 16/10, 117997 Moscow, Russia; mstreltsova@mail.ru (M.A.S.); mashaust1397@gmail.com (M.O.U.); sonya.erokhina@gmail.com (S.A.K.); rodicvelic@gmail.com (R.A.V.); 2 Bristol Myers Squibb, Seattle, WA 98109, USA; barsov@hotmail.com

**Keywords:** NK cells, genetic modification, cancer cell therapy, K562-mbIL21, retroviral vectors, transduction, hTERT, telomerase, telomerase activity

## Abstract

NK cells are the first line of defense against viruses and malignant cells, and their natural functionality makes these cells a promising candidate for cancer cell therapy. The genetic modifications of NK cells, allowing them to overcome some of their inherent limitations, such as low proliferative potential, can enable their use as a therapeutic product. We demonstrate that hTERT-engineered NK cell cultures maintain a high percentage of cells in the S/G2 phase for an extended time after transduction, while the life span of NK cells is measurably extended. Bulk and clonal NK cell cultures pre-activated in vitro with IL-2 and K562-mbIL21 feeder cells can be transduced with hTERT more efficiently compared with the cells activated with IL-2 alone. Overexpressed hTERT was functionally active in transduced NK cells, which displayed upregulated expression of the activation marker HLA-DR, and decreased expression of the maturation marker CD57 and activating receptor NKp46. Larger numbers of KIR2DL2/3+ cells in hTERT-engineered populations may indicate that NK cells with this phenotype are more susceptible to transduction. The hTERT-modified NK cells demonstrated a high natural cytotoxic response towards K562 cells and stably expressed Ki67, a proliferation marker. Overall, our data show that ectopic hTERT expression in NK cells enhances their activation and proliferation, extends in vitro life span, and can be a useful tool in developing NK-based cancer cell therapies.

## 1. Introduction

Natural killer (NK) cells constitute the first line of defense against viruses and tumors. They recognize altered and infected cells with a reduced surface expression of MHC I, and cells with increased expression of stress molecules and pathogen-associated molecular patterns via a set of activating and inhibiting receptors [1]. Due to their high cytotoxic potential and natural ability to recognize tumors through a wide spectrum of receptors, NK cells can be considered as a promising candidate cell type for cancer immunotherapy. Genetically modified NK cells can provide several key advantages over engineered T cells as therapeutic agents. Specifically, NK cells are unable to cause graft versus host disease [2], and their antitumor activity may be further enhanced by combining with therapeutic antibodies, chemotherapeutic agents, and radiation [3,4].

Numerous studies targeted enhancing NK cell expansion and engineering them genetically, striving to improve their desired functional characteristics. Based on recent advances in understanding the regulation of NK cell activity, genetic engineering of NK cells is expected to help unlock their full potential for immunotherapy. Despite the advantageous safety profile, the efficacy of NK cells in human trials remains poor, especially for solid tumor indications. Several inherent properties of NK cells restrict their use in cancer immunotherapy [5]. In particular, NK cells have rather limited capability to expand both in vitro and in vivo [6,7,8], with an estimated half-life of major NK subsets in humans to be less than ten days [9]. The inability to expand beyond naturally modest total cell numbers curtails the maximum-achievable dose of the cells during ex vivo engineering and expansion while decreasing the longevity of adoptively transferred engineered NK cells in patients.

The activation of NK cells followed by their long-term in vitro expansion leads to replicative senescence, which results from a loss of integrity and length of telomeres. Telomere loss negatively affects the proliferative capacity of the cells via genome-wide epigenetic changes in the chromatin and associated modulation of transcriptional activity in key regulatory circuits. Endogenous telomerase activity in NK cells is typically insufficient to support the continuous restoration of telomeres, which are shortened with each cell division [10]. One way to overcome this problem would be to ectopically express the catalytic subunit of human telomerase reverse transcriptase (hTERT) in NK cells. Overexpression of hTERT, a rate-limiting component of the telomerase complex, could be expected to compensate for an insufficient amount of endogenous enzyme, thus protecting the cells from replicative senescence [11]. Healthy donor-derived NK cells transduced with a retroviral vector expressing hTERT had a longer lifespan in cell culture compared to the control cells [12]. However, retroviral transduction of NK cells is typically difficult and less efficient in comparison to the other cells of hematopoietic lineage. This is attributed to the fact that NK cells as the first responders to viral infections [13] are designed and evolutionarily selected to be highly resistant to viral penetration [14] during natural infections. Accordingly, NK cells cannot be efficiently transduced with viral vectors. To increase the efficiency of transduction, it is typically necessary to carry out some additional manipulations with NK cells, such as in vitro pre-stimulation of the cells [15].

IL-2 is one of the key growth factors supporting the expansion of NK cells and increasing the transduction efficiency. However, IL-2 alone is insufficient, and for achieving significant activation and expansion of these cells in culture, additional cofactors (such as IL-21) or stimuli (IL-21-engineered feeder cells) are normally required. In comparison with soluble IL-21, K562 cells engineered with membrane-bound IL-21 (K562-mbIL21) were shown to induce a substantial increase in the number of NK cells in co-culture in the presence of IL-2 [16]. These observations clearly suggest that the expansion of NK cells and the efficiency of viral vector-mediated gene delivery to them could be increased by including membrane-bound cytokines such as IL-21 in the ex vivo culture conditions.

In this study, we compared the efficiency of gene delivery for various subsets of freshly isolated NK cells, as well as for bulk and clonal cultures that were activated in vitro in the presence of IL-2 and K562-mbIL21 feeder cells before transduction. We evaluated the enzymatic activity of ectopically expressed hTERT and studied the effect of transduction on the phenotypic and functional properties of NK cells. Our results demonstrate a substantial increase in proliferative activity and expansion of NK cells caused by hTERT overexpression, which prompted us to suggest that hTERT-engineered NK cells could become a valuable tool in the cancer cell therapy toolbox.

## 2. Materials and Methods

### 2.1. Cell Lines

The erythroblastic leukemia cell line K562 was obtained from ATCC (Manassass, VA, USA). The genetically modified K562 clone expressing membrane IL-21 (K562-mbIL21) was kindly provided by Dean Lee (MD Anderson Cancer Center, Houston, TX, USA). In addition to mbIL-21, this clone also expresses CD64, CD86, CD137L, and the fragment of the CD19 receptor [17]. GP2-293 retroviral packaging cell line expressing gag and pol genes, a derivative of human embryonic kidney HEK293T cells (Clontech/Takara, Terra Bella Ave. Mountain View, CA, USA), was used for vector packaging and preparation of high titer retroviral vector stocks. GP2-293 cells were cultured in a DMEM medium (PanEco, Russian Federation) supplemented with 10% FCS, 2 mM L-glutamine, 2 mM sodium pyruvate (PanEco, Russian Federation), 2 mM antibiotic-antimycotic (Sigma-Aldrich, St. Louis, MO, USA). K562 and K562-mbIL21 cell lines were maintained in a RPMI-1640 medium (PanEco, Russian Federation) supplemented with 10% fetal calf serum (FCS, HyClone Labs, Logan, UT, USA), 2 mM L-glutamine (PanEco, Russian Federation), and 2 mM antibiotic-antimycotic at a concentration of 2–6 × 10^5^ cells/mL. The surface expression of IL-21 in K562-mbIL21 cells was periodically verified by flow cytometry using anti-IL-21-PE antibodies (clone 3A3-N2, BioLegend, San Diego, CA, USA). To prepare feeder cells, K562 or K562-mbIL21 cells were γ-irradiated at 100 Gy, frozen, and stored at –135 °C.

### 2.2. Isolation, Activation, and Culture of NK Cells

Blood samples were obtained from healthy individuals of different gender and age. All participants gave verbal informed consents prior to the study, which was approved by the local ethics committee (Pirogov Russian National Research Medical University). Peripheral blood mononuclear cells (PBMC) were obtained by gradient centrifugation on a 1.077 g/mL Ficoll gradient (PanEco, RF). NK cells were isolated from PBMC by negative magnetic separation using an NK cell isolation kit (Miltenyi Biotech, Bergisch Gladbach, Germany) according to the manufacturer’s protocol. The purity of isolated cells was typically at least 97%.

Freshly isolated NK cells were grown in 24-well plates in NK MACS Medium (Miltenyi Biotech, Bergisch Gladbach, Germany) supplemented with 100 U/mL IL-2 (Hoffmann La-Roche, Basel, Switzerland) and irradiated K562-mbIL21 feeder cells at an NK: feeder ratio of 4:5. The cells were cultured at 37 °C with 5% of CO_2_. After six days in culture, the medium was partially replaced with a fresh one, the concentration of IL-2 was adjusted to 100 U/mL, and the cells were transferred to the new 24-well plate and grown for another 4–5 days before transduction.

### 2.3. Generation of NK Cell Clones

The FACSVantage DiVa cell sorter (Beckton Dickinson, Franklin Lakes, NJ 07417, USA), equipped with 405, 488, and 643 nm lasers and a corresponding set of detectors and filters, was used for cell sorting. To generate clonal NK cell cultures, freshly isolated cells were pre-stained with fluorochrome-conjugated monoclonal antibodies against CD56 and CD3, and sorted into 96-well round-bottom plates at one cell per well, in the “single cell” cell sorter mode [18]. At least 120 cells were sorted. Plates prepared for cell deposition contained K562-mbIL21 feeder cells at a concentration of 104 cells per ml in the complete cloning medium (DMEM supplemented with 20% ExVivo medium (Thermo Fisher Scientific, San Jose, CA, USA) and 100 U/mL recombinant human IL-2 (Hoffmann La-Roche, Basel, Switzerland). Half of the spent medium was replaced one time after three weeks of incubation at 37 °C (5% CO_2_), and then once weekly. The clones that attained the cell number of 2 × 10^5^ cells were expanded to 24-well plates and maintained at a concentration of 0.5 × 10^6^ cells/mL. In clonal cultures with low proliferative activity, the medium was changed weekly, while the medium in the cultures with good growth was changed as required to maintain the cultures in a healthy state.

### 2.4. Antibodies Used for Flow Cytometry and Cell Sorting

The following mouse monoclonal antibodies were used: CD56-APC (clone N901, Beckman Coulter, Miami, FL, USA), CD56-Brilliant Violet 421 (clone HCD56, Sony Biotechnology, San Jose, CA, USA), CD56-PE (clone N901 (HLDA6), Beckman Coulter, Miami, FL, USA), CD57-PE (clone TB01, eBioscience, San Diego, CA, USA), CD57-FITC (clone TB03, Miltenyi Biotec, Bergisch Gladbach, Germany), CD57-APC (clone TB03, Miltenyi Biotec, Bergisch Gladbach, Germany), CD16-PE (Sorbent, RF), CD2-PE-Cy7 (clone TS1/8, Sony Biotechnology, San Jose, CA, USA), anti-NKG2A-PE (clone 131411, R&D Systems, Minneapolis, MN, USA), anti-KIR2DL2/DL3-PE (clone DX27, Miltenyi Biotec, Bergisch Gladbach, Germany), anti-HLA-DR-FITC (clone B8.12.2, Beckman Coulter, Miami, FL, CA, USA), NKG2A-PE (clone 131411), NKG2C-AlexaFluor488 (clone 108724), NKG2C-PE (clone 134591; R&D Systems, Minneapolis, MN, USA), NKp46-FITC (clone 9E2; Sony Biotechnology, San Jose, CA, USA), anti-NKG2D-PE (clone REA1175, Miltenyi Biotec, Bergisch Gladbach, Germany). Staining was performed according to the manufacturer’s instructions.

### 2.5. Flow Cytometry

A MACSQuant 10 cytometer (Milteniy Biotech, Bergisch Gladbach, Germany) equipped with 405 nm, 488 nm, and 635 nm lasers was used for the acquisition of flow cytometry data. The results were processed using Flowing Software version 2.5.1 (Perttu Terho, Turku Center for Biotechnology, Turku, Finland) or FlowJo software version 7.6 (TreeStar Williamson Way, Ashland, OR, USA). Statistical analysis was performed using SigmaPlot 12 (SYSTAT Software Inc., Washington St, Chicago, IL, USA) and GraphPad Prism 7 (StatSoft Inc., Tulsa, OK, USA) software.

### 2.6. NK Cell Degranulation Assay

To estimate the level of effector cell degranulation, K562 cells were mixed with NK cells at a 1:1 ratio in the culture medium supplemented with brefeldin A (10 μg/mL, Invitrogen, San Jose, CA, USA) and anti-CD107a antibody (Milteniy Biotech, Germany). The samples were incubated for 2.5 h at 37 °C (5% CO_2_), followed by washing to remove unbound antibodies and staining for surface markers. The surface levels of CD107a on NK cells were quantified using the MACSQuant 10 flow cytometer.

### 2.7. Cell Viability Assay

The analysis of cell viability was performed by staining cells with SYTOXRed Dead Cell Stain solution (Invitrogen, San Jose, CA, USA) for 2–5 min in the dark. Alternatively, staining with fluorescent annexin V conjugate (Invitrogen, San Jose, CA, USA) was performed for 15 min at room temperature, followed by adding the propidium iodide (PI, Sigma-Aldrich, St. Louis, MO, USA) solution (2 μg/mL).

### 2.8. Analysis of Cell Cycle with PI DNA Staining

The NK cell cycle analysis was performed by quantification of DNA amount per cell with PI staining. The cells were harvested, washed in the phosphate-buffered saline (PBS), and fixed in cold 70% ethanol for 1 h, at −4 °C. Subsequently, the cells were centrifuged at 300× *g* for 10 min, washed with PBS, treated with RNAse (Thermo Fisher, San Jose, CA, USA), and stained with PI according to a standard protocol. Stained cells were analyzed by flow cytometry using MACSQuant 10.

### 2.9. Analysis of Proliferative Activity

The proliferation of NK cells was evaluated by measuring the percentage of Ki67+ cells in cultures. The cells were harvested, washed in PBS, stained by fluorescent antibodies against CD56 and CD57 for 25 min, washed in PBS, and fixed in cold 70% ethanol for 1 h, at −4 °C. Fixed cells were centrifuged at 300× *g*, rinsed by PBS, and stained by fluorescent antibodies against Ki67. Stained NK cells were washed in PBS and analyzed in MACSQuant 10 flow cytometer.

### 2.10. Cell Counting

NK cells were counted by automatic cell counter TC20 (Bio-Rad, Hercules, CA, USA) using the manufacturer-recommended protocol.

### 2.11. Retroviral Transduction

The hTERT cDNA was fused to the 3’ terminus of the GFP DNA via P2A self-cleaving peptide for bi-cistronic expression [19]. Retroviral vector xlox-GFP-hTERT expressing GFP-P2A-hTERT fusion protein was used for NK cell transduction (plasmids were kindly provided by Frederick National Laboratory for Cancer Research, Frederick, MD, USA) [20]. The vector was pseudotyped with RD114 envelope glycoprotein to maximize transduction efficiency [21]. Pure plasmid DNA was isolated by using GeneJET Plasmid Maxiprep Kit (ThermoFisher Scientific, San Jose, CA, USA) according to the manufacturer’s instructions. High titer retroviral vector stocks were prepared by co-transfecting GP2-293 cells with xlox-GFP-P2A-hTERT DNA and RD114 expression construct in 100 mm Petri dishes treated with poly-L-lysine solution (Sigma-Aldrich, St. Louis, MO, USA), using a calcium phosphate transfection kit (ThermoFisher Scientific, San Jose, CA, USA) according to the manufacturer’s instructions. The cell culture medium containing packaged vector was harvested at 48 and 72 h post-transduction, filtered through Millex-HV-0.45 µm PES filter (Millipore, Burlington, MA, USA), and concentrated on sterile cones with 100 nm pores (Millipore, Burlington, MA, USA). The transduction of bulk and clonal NK cells was performed in 24-well plates treated with a Retronectin solution (Clontech/Takara, Terra Bella Ave. Mountain View, CA USA) in PBS at a final concentration of 20 μg/mL, according to manufacturer’s recommendations. The transduction efficiency was measured as a percentage of GFP+ NK cells in 3–5 days after the transduction.

### 2.12. Immunophenotyping of GFP-P2A-hTERT-Transduced NK Cells

The surface expression of NK-specific markers was determined by flow cytometry analysis of untransduced and transduced NK cells. Since the transduction efficiency in vector-infected cultures never reached 100%, an untransduced fraction of NK cells (GFP-hTERT-) was used as a convenient internal reference. For this, NK cells in vector-infected cultures were stained with appropriate surface marker antibodies and gated on GFP positivity to discriminate GFP-positive transduced (hTERT+) and GFP-negative (hTERT−) cells. The surface marker expression was then measured in each fraction and compared between fractions.

### 2.13. Evaluation of Telomerase Activity

The NK cells in transduced and untransduced cultures were counted. To evaluate and compare hTERT enzymatic activity between these cultures, all samples were normalized to contain equal cell numbers per volume. Nuclear extracts were prepared from samples, each containing an equal number of NK cells. Enzymatic activity of hTERT was measured in nuclear extracts by telomere repeat amplification protocol (TRAP) [22] using TRAPeze Telomerase Detection Kit (Millipore, Burlington, MA, USA), according to the manufacturer’s instructions. Telomerase activity was evaluated by visual comparison of width and number of telomere repeat DNA bands with positive control provided in the kit.

## 3. Results

### 3.1. Delivery of hTERT into Activated NK Cells by Retroviral Transduction

**Figure 1:**
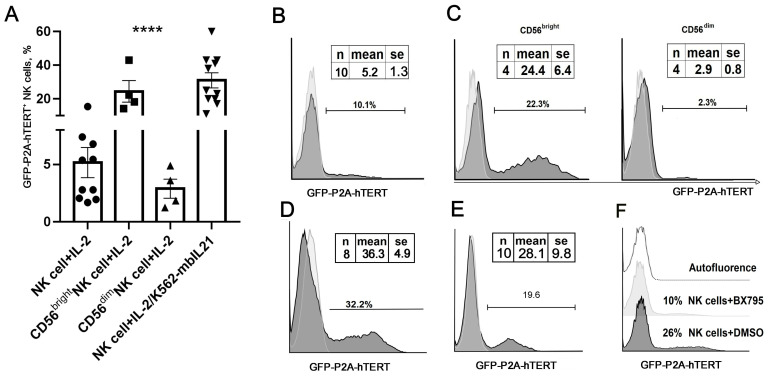
Engineering of ex vivo activated NK cells with a retroviral vector expressing GFP-P2A-hTERT gene. (**A**) Comparison of the transduction efficiency in bulk and subset NK cell cultures activated by two different means. Statistical differences were calculated using one-way ANOVA (**** *p* < 0.0001). The transduction efficiency of various NK culture types is marked as follows: circles-bulk NK cells cultured with IL-2, squares - CD56^bright^NK cells grown with IL-2, up triangles - CD56^dim^NK cells cultured with IL-2, down triangles - bulk NK cells grown with IL-2+K562-mbIL21. (**B**–**D**) Representative histograms of GFP-P2A-hTERT transduction efficiency of: (**B**) bulk population of NK cells activated by IL-2 for seven days; (**C**) CD56^bright^ (left) and CD56^dim^ (right) subsets sorted and activated by IL-2 for 7 days; (**D**) bulk population of NK cells activated with K562-mbIL21 and IL-2 for 10 to 12 days. Autofluorescence level is marked with a light gray line. (**E**) Transduction efficiency for the clonal cultures generated from the bulk population of NK cells. A mean average of ten clones is shown. (**F**) The effect of the BX795 inhibitor on the hTERT transduction of NK cells stimulated by the combination of IL-2/K562-mbIL21.

To simplify the detection and quantification of hTERT expression in transduced NK cells and their isolation by FACS, we delivered hTERT cDNA in the cells as a fusion to the GFP through the P2A self-cleaving peptide (GFP-P2A-hTERT), which ensured equimolar expression of both individual proteins from a bi-cistronic retroviral vector [19]. To deliver hTERT into human NK cells, we used previously established retroviral vectors pseudotyped with the envelope protein of endogenous feline retrovirus RD114 [23]. Since gamma-retroviruses and vectors derived thereof can only integrate into the genome of dividing cells, using these vectors to transduce non-dividing freshly isolated NK cells is extremely inefficient [23]. Consequently, in this study, we attempted to transduce the cells that, prior to transduction, were activated by two different stimuli, IL-2 and membrane-bound IL-21. Initially, we attempted to transduce NK cells activated for seven days with IL-2, a cytokine commonly used to induce and support NK cell proliferation. IL-2 mostly and predominantly activates CD56^bright^ cells, as they express high-affinity IL-2 receptors [24]. The efficiency of hTERT transduction in the bulk population of IL-2-activated NK cells was low (5.2 ± 1.3% (hereinafter mean ± SEM is presented); Figure 1A,B). As expected, susceptibility to retroviral transduction of less differentiated CD56^bright^ NK cells, isolated by cell sorting and activated in vitro with IL-2 for a week, was higher in comparison with more differentiated similarly activated CD56^dim^ cells (Figure 1A,C). On average, the transduction of CD56^bright^ NK cells (24.4 ± 6.4%) was four times more efficient than the one of the bulk unseparated NK cell population. At the same time, the average expression level of GFP-P2A-hTERT protein in CD56^dim^ NK cells (2.9 ± 0.8%) was lower than in the whole population. These data suggest that IL-2-mediated activation enhanced the transduction efficiency predominantly in CD56^bright^ NK cells. Of note, CD56^bright^ represents fairly small proportion (5–10%) of peripheral blood-derived NK cells. In addition, according to our earlier observation, most of the expanded NK cell clones in our hands were derived from CD56^dim^CD57^neg^ population [18]. As the low abundance of CD56^bright^ NK cells would make them impractical to be used in adoptive immunotherapy, we did not concentrate our efforts on this population, and instead focused our analyses on the more abundant CD56^dim^CD57^neg^ cells.

We have previously shown that the combination of soluble IL-2 and K562-mbIL21 feeder cells expressing membrane-bound IL-21 promoted retroviral transduction of NK cells, which were generally difficult to transduce [23]. While this activation strategy could be used to support the active proliferation of both CD56^dim^ and CD56^bright^ NK cells, neither sorted CD56^bright^ cells nor clonal lines derived from them expanded well. Therefore, here we stimulated the bulk population of NK cells with membrane-bound IL-21 on irradiated K562-mbIL21 feeder cells in the presence of IL-2 for 10 to 12 days prior to transduction. The transduction efficiency observed in IL-2/K562-mbIL21-stimulated cells (Figure 1A,D) was statistically higher than that observed in the cells stimulated with IL-2 alone (Figure 1B). Similar GFP-P2A-hTERT transduction efficiency was shown for clonal NK cell lines generated by activation with IL-2/K562-mbIL21 as described earlier [15] (Figure 1E). Treatment of the cells with TBK1/IKKε inhibitor BX795, which is known to promote lentiviral gene transduction [25], provided no measurable benefits and even decreased the efficiency of transduction (Figure 1F).

### 3.2. Enzymatic Activity of Telomerase in hTERT-Transduced NK Cells

**Figure 2:**
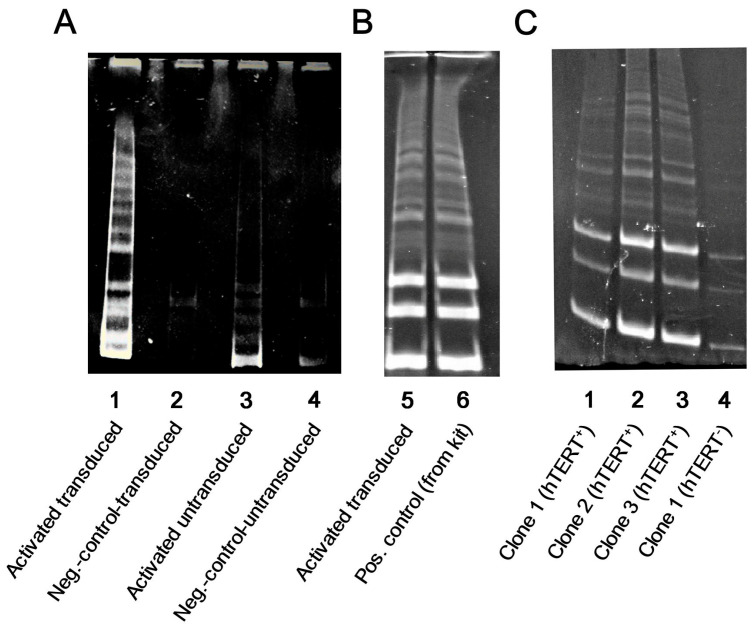
Determination of enzymatic activity of telomerase in GFP-P2A-hTERT-transduced bulk NK cells and clonal lines. (**A**,**B**) Bulk cultures: telomerase activity in transduced (1, 5) and untransduced (3) NK cells. Negative controls (2, 4): nuclear extract from transduced (2) and untransduced (4) NK cells inactivated by heating; 6—standard positive control supplied with the TRAPeze kit. (**C**) Clonal cultures: telomerase activity in transduced (1, 2, 3) and untransduced (4) NK clonal lines.

Having established a protocol for an efficient hTERT transduction of NK cells, we next evaluated the enzymatic activity of telomerase in GFP-P2A-hTERT-transduced cells. For that, the cells were sorted to isolate the GFP-P2A-hTERT-positive population with at least 96% purity. As shown in Figure 2A,B, telomerase activity evaluated by TRAP assay in activated transduced NK cells (lanes 1 and 5) was substantially higher than the baseline endogenous activity in activated untransduced cells (lane 3) and was comparable to the telomerase activity in commercial positive control included with TRAPeze kit (lane 6). Telomerase activity was also increased in transduced clonal NK cell cultures (Figure 2C, lanes 1–3) compared to the background in untransduced ones (Figure 2C, lane 4). Altogether, this confirms that enzymatically active hTERT was ectopically expressed in transduced NK cells from the GFP-P2A-hTERT retroviral vector.

### 3.3. Effects of Ectopically Expressed hTERT on Phenotypic and Functional Characteristics of NK Cells

**Figure 3:**
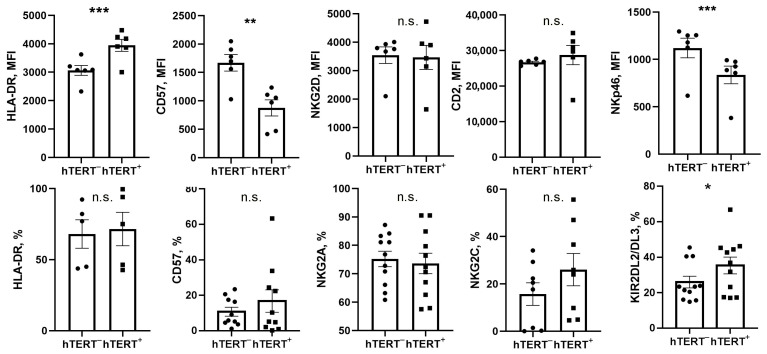
Comparison of the phenotypic characteristics of GFP-P2A-hTERT-positive (hTERT^+^, shown in squares) and negative (hTERT^–^, shown in circles) NK cells in transduced cultures, measured seven days after the transduction. Statistical analysis was performed using paired *t*-test, means ± SEM are presented (* *p* < 0.05; ** *p* < 0.01; *** *p* < 0.001).

To study the effects of ectopically expressed hTERT on NK cell biology, we performed immunophenotyping and functional assays with hTERT-engineered cells seven days after transduction with GFP-P2A-hTERT vector (Figure 3). Since the efficiency of transduction was lower than 100%, each vector-infected NK cell culture contained both transduced (hTERT^+^ GFP^+^) and untransduced (hTERT^–^ GFP^–^) cells. Using the untransduced fractions (hTERT^−^ GFP^−^) cells in each culture as an internal negative control, we found that the expression of the NK cell activation marker HLA-DR was upregulated in GFP-positive hTERT^+^ cell fraction as compared to GFP-negative hTERT^–^ fraction from the same culture (Figure 3). In contrast, we observed a decreased expression of differentiation marker CD57 and natural cytotoxicity receptor NKp46 in transduced NK cells. The GFP^+^ hTERT^+^ and GFP^–^hTERT^–^ cells displayed similar levels of CD2 and NKG2D expression (Figure 3). No significant differences were found in the NKG2A and NKG2C-positive cell percentages in the analyzed fractions. However, in hTERT^+^ cells, a slightly larger population expressed KIR2DL2/3 receptor as compared to hTERT^–^ fraction cells (Figure 3), suggesting that KIR2DL2/3^+^ NK cells either were transduced more efficiently or survived better after transduction.

More detailed analysis of isolated subpopulations may be required to account for the differences in susceptibility to transduction and to clarify an individual response of these subpopulations to hTERT.

### 3.4. Cytotoxic Activity of hTERT-Engineered NK Cells

**Figure 4:**
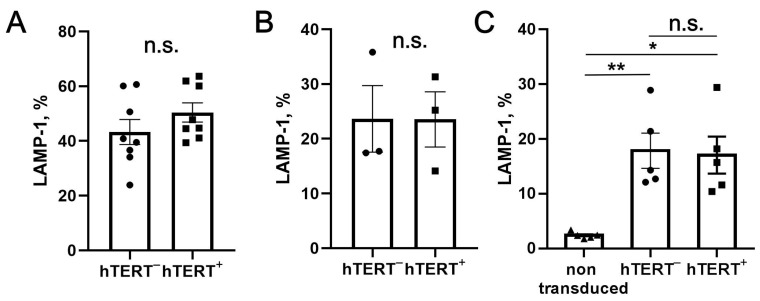
Degranulation response of hTERT-expressing NK cells and clones, measured by LAMP-1 surface level seven days post transduction. (**A**) Degranulation of GFP-P2A-hTERT-positive (hTERT^+^, shown in circles) and negative (hTERT^–^, shown in squares) NK cell fractions in the presence of K562 target cells. (**B**) Degranulation of hTERT-transduced clonal lines in the presence of K562 cells. (**C**) Spontaneous degranulation levels in untransduced cells and GFP-P2A-hTERT^+^ and GFP-P2A-hTERT^–^ cell fractions from transduced cultures. hTERT^–^, hTERT^+^, and spontaneous degranulation levels (non-transduced) are shown in circles, squares, and triangles, respectively. In A and B, the values minus controls are given. Statistical analysis was performed using paired *t*-test. Means ± SEM are presented (* *p* < 0.05; ** *p* < 0.01).

Next, we studied the cytotoxic activity of hTERT-engineered NK cells by using a degranulation assay (Figure 4). In both bulk and clonal transduced cultures, GFP-P2A-hTERT^+^ NK cells displayed similar degranulation activity in response to standard target cells K562, in comparison with GFP-P2A-hTERT^–^ controls (Figure 4A,B), while showing a higher level of spontaneous degranulation compared to untransduced NK cell cultures (Figure 4C).

### 3.5. Survival and Proliferative Activity of hTERT-Engineered NK Cells

**Figure 5:**
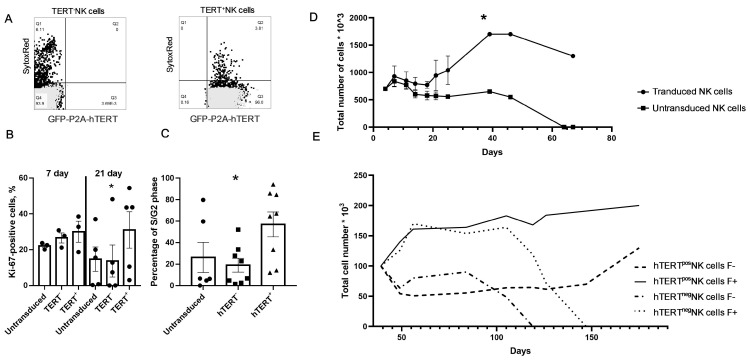
Comparison of the viability and proliferation of GFP-P2A-hTERT-transduced and untransduced NK cells. Statistical differences were calculated using one-way ANOVA (* *p* < 0.05). (**A**) Representative data of the viability of untransduced (left) and GFP-P2A-hTERT-transduced (right) NK cells. Light gray shade—autofluorescence, dark gray shade—cells stained with SYTOX RED. (**B**) Intracellular Ki67 expression levels in untransduced cells and GFP^–^ hTERT^–^ (hTERT^–^) and GFP^+^ hTERT^+^ (hTERT^+^) NK cells from GFP-P2A-hTERT vector-infected cultures on days 7 and 21 after the transduction. Average results of five independent experiments are presented. (**C**) Phases of the cell cycle in untransduced and GFP-P2A-hTERT-transduced GFP^–^ hTERT^–^ cells (hTERT^–^) and GFP^+^ hTERT^+^ (hTERT+) NK cell fractions in retroviral vector - infected cultures 21 days post transduction. Average data from eight individual experiments are presented. Circles indicate percentage of untransduced NK cells, squares – percentage of GFP^–^ hTERT^–^, and triangles – percentage of GFP^+^ hTERT^+^ cells in S/G2 phase of the cell cycle. (**D**) Cell numbers of untransduced or transduced NK cells cultured in the presence of IL-2 (average data from three experiments). (**E**) Cell numbers in the cultures of sorted hTERT-positive and hTERT-negative NK cells grown in the presence of IL-2 (F–), or with a combination of IL-2 and K562-mbIL21 feeder cells (F+).

Finally, we asked whether hTERT overexpression affected the proliferative activity and lifespan of cultured NK cells. Initially, we looked at the viability of the cells cultured with IL-2 alone, without additional stimuli. Both GFP-P2A-hTERT-positive and GFP-P2A-hTERT-negative NK cells from transduced cultures were highly viable one-week post transduction: the percentage of necrotic cells for transduced cells was 3% ± 1.1 versus 5.7% ± 0.4 for unmodified cells (mean ± SE; Figure 5a).

We also evaluated the proliferative activity of NK cell cultures by intracellular staining for Ki67 at days 7 and 21 post transduction. No differences in Ki67 expression were seen in the cultures on day 7 (Figure 5B). The mean values were 22.1 ± 1% for untransduced control cultures, 26.6 ± 2.8% for TERT^–^ cell fractions, and 30 ± 5.9% for hTERT^+^ fractions from transduced NK cell cultures. By day 21, Ki67 expression went down in both types of control cells, and the percentage of Ki67^+^ cells in hTERT^+^ fractions remained at the same level (31 ± 10.1%; Figure 5B). Cell cycle analysis showed that 63 ± 10.6% of GFP^+^ hTERT^+^ cells were in phases S and G2 three weeks after transduction, while only 21 ± 6.6% of GFP^–^TERT^–^ NK cells were proliferating. In the untransduced activated cell cultures, 30 ± 15.1% of cells were in S/G2 phase (Figure 5C).

Further, we studied cell number kinetics and the lifespan of hTERT-engineered cells. First, we compared hTERT-transduced and untransduced cells growing in the same conditions (Figure 5D). While similar cell number kinetics was observed for these cultures in the first two weeks, the longer-term cultures of hTERT-transduced cells reached higher cell numbers in comparison with untransduced controls. The lifespan of untransduced control cells was limited to approximately two months, while hTERT-transduced cells kept proliferating and expanding for about one month longer. These data prompted us to suggest that ectopic expression of hTERT was responsible for promoting proliferation and increasing the lifespan of NK cells. Importantly, while surviving ex vivo much longer (more than 70 days) than untransduced controls, hTERT-transduced cells still did not expand beyond 40 to 50 days of culture (Figure 5D). This suggested that, while increasing the overall longevity of NK cells, ectopic hTERT expression was not sufficient to induce true immortalization, for which some additional factors may have been required.

Next, we suggested that stimulation with K562-mbIL21 feeder cells could further increase the lifespan of hTERT-engineered cells. As shown in Figure 5B,C, in the presence of IL-2 alone, the cells actively proliferated at day 21 post transduction, as determined by Ki67 expression and cell cycle analysis. Based on this, we analyzed the effect of K562-mbIL21-mediated activation on the survival and proliferative activity of hTERT-transduced NK cells in long-term cultures. GFP-P2A-hTERT-positive and negative cells were isolated by cell sorting and were cultured in the presence of IL-2 at 100 U/mL for 35 days. Subsequently, the cultures of each type were split into two parts, which were further cultured either with IL-2 alone or in the presence of IL-2 and K562-mbIL21 feeder cells, at a 4:5 ratio of NK:K562-mbIL21 (Figure 5E). Both hTERT^+^ and hTERT^–^ cells cultured with feeder cells grew well for two weeks, whereas the cells cultured with IL-2 alone did not grow. Subsequently, the growth rate of both hTERT^+^ and hTERT^–^ cells activated by K562-mbIL21 decreased, which was possibly associated with the depletion of live feeder cells. TERT^–^ cells cultured without feeder cells and those stimulated by K562-mbIL21 died after 119 and 147 days, respectively, while hTERT-positive cells continued to expand, with higher cell count in the cultures stimulated by feeder cells. Thus, the addition of feeder cells post transduction supported the proliferation of NK cells, which allowed hTERT-engineered cells to expand and survive longer than the untransduced control cells. Moreover, activation by K562-mbIL21 cells and ectopic hTERT expression appeared to act synergistically on increasing NK cell proliferation and life span.

## 4. Discussion

Genetic engineering of NK cells toward creating better products for cancer cell therapy is becoming an increasingly widespread approach. A number of important questions pertinent to engineering of these cells with retroviral and lentiviral vectors remain unresolved. As the retroviral/lentiviral transduction of NK cells is subpar in efficiency in comparison to T cells, it is still unclear what conditions would make NK cells more susceptible to these vectors. In particular, the best methodology for pre-transduction activation of NK cells is not yet established. In this study, we have shown that, although the activation of NK cells by IL-2 is necessary, it alone is not sufficient to achieve high transduction efficiency. This may be caused by the low proliferation activity of IL-2-stimulated NK cells, since active proliferation is a pre-requisite for gamma-retroviral vector integration [26]. Circulating NK cells are a heterogeneous population of lymphocytes, some of them being less sensitive to IL-2 [27], which is reflected in the total level of activation and proliferation in bulk NK populations. NK cells with CD56^bright^ phenotype demonstrate greater proliferative activity under IL-2 stimulation compared to CD56^dim^ cells, due to expressing high-affinity IL-2 receptors (in some cases, at a higher density) [28]. In our study, we observed better efficiency of CD56^bright^ NK cell transduction in comparison with CD56^dim^ cells, which corresponds to a higher proliferative activity of the CD56^bright^ subset upon IL-2 stimulation. However, a low percentage of CD56^bright^ subset (rarely exceeding 10–15% in peripheral blood NK cells), typically seen in healthy individuals [24,29], makes producing therapeutically adequate numbers of these cells impractical. In addition to CD56^dim^ cells, another NK cell subset (CD57^+^) is difficult to transduce, as most of the cells in this subset are in the G0 phase of the cell cycle [23].

We looked for some additional stimulation conditions that could increase the transduction efficiency of bulk NK populations. Previous work demonstrated that stimulation of NK cells with K562-mbIL21 feeder cells leads to a greater expansion of NK cells in vitro than stimulation with K562-mbIL15 feeders [17]. In our laboratory, using clonal cultures of NK cells, we showed that K562-mbIL21 cells in combination with IL-2 promoted active proliferation of both CD56^bright^ and CD56^dim^ subsets. The largest expansion was observed in the clones derived from CD56^dim^ NK cells negative for CD57, the replicative senescence marker [15]. This method of combined mbIL-21 and IL-2 stimulation was recently successfully applied to generate NK cells expressing CAR [30].

Retroviral vectors can integrate into virtually any genomic locus, carrying the risk of insertional mutagenesis and subsequent transformation. If the genes involved in cell cycle regulation are destroyed by vector insertion, or genes responsible for apoptosis and survival are activated, down-regulation of proliferation can potentially occur in transduced cells, which may then die post transduction. Some other insertional mutagenesis events may induce cell division, thus conveying proliferative advantage to the transduced cells. Accordingly, immunophenotyping of transduced primary cells is best performed at least a week post transduction, to give the cells carrying insertional mutagenesis-activated or destroyed genes the time to expand or to die.

As we have shown here, hTERT-transduced cells showed an increased expression of the activation marker HLA-DR, while displaying higher proliferative activity. Decreased CD57 expression on the surface of transduced NK cells is also characteristic of more rapidly dividing cells. NK cells with higher proliferative activity are expected to be more susceptible for the GFP-P2A-hTERT retroviral transduction, as we reported earlier [23]. Alternatively, this may be attributed to post-transduction selection of better proliferating cells due to insertional mutagenesis. Finally, overexpression of hTERT in transduced NK cells may lead to an increase in HLA-DR expression and a decrease in CD57 levels. The observed increase of the KIR2DL2/DL3^+^ population in transduced cells can also be explained by its increased rate of division, as this population possesses a more differentiated phenotype, proliferates faster, and is more efficiently transduced. It is also possible that the increase in the percentage of KIR2DL2/DL3-positive cells occurred due to their better survival or enhanced proliferation. According to earlier work, KIR expression is a relatively stable characteristic of cultured NK cells [18,31]. Thus, while it cannot be completely ruled out, it seems less likely that an increase in the percentage of KIR2DL2/DL3^+^ NK cells after transduction occurs due to the induction of these receptors during long-term culture. Rather, it may be a direct consequence of hTERT-stimulated proliferation. The higher percentage of more-differentiated KIR2DL2/DL3-positive fraction in the total population of transduced cells can potentially lead to an increased degranulation in hTERT-engineered NK cells compared with control unmodified cells. However, we did not detect a significant difference in the degranulation activity between GFP-P2A-hTERT-positive and negative NK cells.

Ectopic expression of hTERT enables cells to overcome the Hayflick limit by allowing more cell divisions and preventing growth arrest [32]. However, hTERT-induced unlimited proliferative potential is not necessarily directly linked with the modulation of the proliferation rate. Rather, hTERT prevents cells from reaching senescence via continuous restoration of telomeres, as well as through other non-telomeric-related mechanisms (e.g., interfering with apoptosis). Various effects of hTERT unrelated to telomerase activity (induction of growth factor receptor expression, alterations in epigenetic and apoptotic mechanisms, signal transduction, energy metabolism, and protein synthesis [33]) have been described for several cell types. It was demonstrated that overexpression of hTERT in cancer cells could decrease intracellular ROS production, thus reducing ROS-mediated apoptosis [34]. A genome-wide study also showed that hTERT ubiquitously bound to regions that encode tRNAs and RNA Pol III subunit RPC32, enhancing tRNAs expression and promoting increased protein synthesis and cell growth [35]. To what extent the non-telomeric activities of overexpressed hTERT could have contributed to the hTERT effect on physiology, proliferation, and life span of NK cells observed in our work, is not clear. While being out of scope for this study, these activities in the context of NK biology certainly warrant further exploration, as they may be intricately involved in the NK cell life span extension that we observed. Studies with catalytically inactive hTERT mutants will be of great interest and importance, especially when considering the safety of hTERT-engineered NK cells in cell therapy settings.

Of note, constitutive hTERT expression is linked to immortality in some cell types (e.g., stem or cancer cells), while only extending the life span beyond physiological limit in the others (e.g., hTERT-engineered somatic primary cells). In this study, we observed substantial enzymatic activity of hTERT in engineered NK cells, which was above the physiologic levels. However, while moderately extending their life span, hTERT overexpression failed to induce limitless division capacity and true immortalization. This is consistent with earlier observations showing that the lifespan of hTERT-NK cells in two donors out of four exceeded the control values by only 1.33 and 1.5 times [12]. In our work, the average lifespan of NK cells engineered with hTERT turned out to be somewhat lower, possibly due to the differences in culture conditions between the studies. Initially, we stimulated transduced cells with IL-2 alone, while Fujisaki et al. used a combination of K562-mb15-41BBL feeder cells and IL-2 [12]. By adding of K562-mbIL21 feeder cells, we were able to further prolong the expansion of hTERT-NK cell cultures. It should also be noted that, as we have previously shown, the weekly addition of feeder cells leads to a decrease in the cytotoxic activity of NK cells, potentially due to the depletion of their degranulating ability [15]. Thus, to create an optimal microenvironment that promotes the accumulation of genetically engineered NK cells with a high cytotoxic potential, further fine-tuning of culture conditions and testing of additional feeder-based culture protocols will be required.

One important aspect of using hTERT-engineered NK cells in cell therapy is safety, as the inability of ectopically expressed hTERT to cause oncogenic transformation of NK cells has not been firmly established so far. In our experiments with NK, we observed an extension of the natural life span rather than complete immortalization; the fact that the effect of hTERT on NK survival and longevity ex vivo is limited may open the way for the use of wild type (or mutant) hTERT to engineer NK-based cell therapeutics. However, further work is required to establish the safety and efficacy of hTERT-modified NK cells as a new generation anti-cancer drug.

## 5. Conclusions

The catalytic subunit of human telomerase (hTERT) was overexpressed in bulk populations and clonal cultures of human NK cells using retroviral transduction. Transduction conditions were optimized to enhance the efficiency by activation of the cells with IL-2 and K562-mbIL21 feeder cells. Overexpression of hTERT modestly affected NK cell phenotype and degranulation, increased proliferative potential, and extended the lifespan of the transduced NK cell cultures, but did not result in a true immortalization. hTERT-mediated NK cell life span extension deserves further investigation and provides a potential resource for improving NK cell-based cancer cell therapies.
